# Experimental study on thermal effect and gas release laws of coal-polyurethane cooperative spontaneous combustion

**DOI:** 10.1038/s41598-021-81537-5

**Published:** 2021-01-21

**Authors:** Haiyan Wang, Yao Tian, Jinglei Li, Xiao Chen

**Affiliations:** School of Emergency Management and Safety Engineering, China University of Mining and Technology (Beijing), Beijing, 100083 People’s Republic of China

**Keywords:** Coal, Composites

## Abstract

This paper presents a research on the kinetics and gas release laws of the cooperative spontaneous combustion of coal-polyurethane binary system by means of thermogravimetric analysis and spontaneous combustion simulation experiments. The coal-polyurethane binary system is more prone than individual samples to combustion. The polyurethane mass fraction has a great influence on the cooperative spontaneous combustion process. As the polyurethane mass fraction rises, the activation energies of oxygen absorption stage and pyrolysis stage decrease. The combustion residues of coal-polyurethane binary system scorches into lumps and the surfaces of the particles become rougher. During spontaneous combustion, the initial release temperatures of the four gases CO_2_, CO, C_2_H_6_ and C_2_H_4_ drop as the polyurethane mass fraction increases. The gas release amount of them increases with increasing polyurethane mass fraction. And the amount of CH_4_ decreases as the polyurethane mass fraction increases. The CO/CO_2_ ratio with temperature for different polyurethane mass fraction have the same trends. The results of this study have implications concerning polyurethane materials application and fire protection in coal mine.

## Introduction

In coal mine areas, spontaneous combustion of coal is one of the greatest catastrophes that disturbs safe mining^1–4^. And polyurethane is a widely used organic polymer material. It is a lightweight material characterized by high strength, high adhesion, safety and efficiency of use. In coal mine production, polyurethane is mainly used to strengthen broken coal and rock layers, fill large falling areas, construct sealed partition walls for goafs and block air leakages^5–7^. During the polyurethane grouting process, the reaction of isocyanate with polyhydric alcohol releases a large amount of heat, which causes the temperature to rise rapidly. This excessive temperature will cause the pyrolysis and combustion of polyurethane and coal. In recent years, there have been many fire accidents caused by the reinforcement and filling of polyurethane materials^8–11^. Furthermore, the oxidation and spontaneous combustion of coal may lead to the pyrolysis and combustion of the polyurethane left in goafs. And a large amount of heat and fire gases will release during these process. In coal mine production, gas monitoring of goafs is an important measure to prevent spontaneous combustion and fire accidents. The cooperative spontaneous combustion of coal-polyurethane binary system have significant impacts on the concentrations of the main fire gases in goafs, which will seriously interferes the monitoring of fire gas. In 2020, the National Coal Mine Safety Administration of China promulgated the Measures for the safety management of reactive polymer materials in coal mines to regulate the useage of polyurethane grouting materials.

Scholars in China and abroad have performed many studies on spontaneous coal combustion mechanisms and gas monitoring. The thermogravimetric analysis (TGA) method has been widely used to research the coal spontaneous combustion mechanism and division of stages therein^12,13^. Chen explained the mechanism of coal pyrolysis and spontaneous combustion and proposed a model for particle fragmentation and volatile diffusion during coal pyrolysis^14^. Deng used the thermogravimetric-Fourier transform infrared (TG-FTIR) method to research the mechanism and ignition temperature of the spontaneous combustion of different kinds of coal. The experiments were designed to investigate the change laws of the temperature, fire gas concentrations and heat intensity of spontaneous combustion^15,16^. In actual production, CO, CO_2_, C_2_H_6_, C_2_H_4_, C_2_H_2_ and other index gases are usually used to judge the situation of coal spontaneous combustion^17,18^. Lu conducted experimental research on the gas release laws of the spontaneous combustion of different kinds of coal based on three factors: temperature, water content and flame retardants. He used CO and C_2_H_4_ as index gases to judge the spontaneous combustion situation of coal^19^. Zhu researched the relationship between the oxygen consumption rate and temperature during the low-temperature spontaneous combustion stage of coal. And he obtained the critical temperatures of coal spontaneous combustion and the fire gas release laws^20^.

Polyurethane is an organic polymer material that contains many additives. It is flammable but will not ignite spontaneously at room temperature. There are a lot of CO, CO_2_ and many toxic gases, such as CO and HCN, released during the combustion of polyurethane^21^. Many scholars researched the mechanisms of pyrolysis and combustion and the flame retardancy modification of polyurethane^22–25^. Polyurethane begins to pyrolyse from 200 °C. During its pyrolysis process, first, formate groups decompose into isocyanate segments and polyols. Then, the polyols decompose into ethers and alcohols with increasing temperature. From 300 to 500 °C, the residues decompose into amines, ethers, volatiles and CO_2_^26^. Oxygen has a significant influence on the pyrolysis and combustion of polyurethane^23,27^. The distribution of the pyrolysis products of polyurethane is determined by temperature, and the primary and secondary pyrolysis products are generated by the breaking of urethane bonds and hydrogen conversion of polyhydric alcohol^28^. Hobbs established a detailed model of the bond breaking and gas product generation mechanism of polyurethane pyrolysis based on TGA experiments, percolation theory, vapor pressure and partial pressure of volatiles^29^. Duquesne measured the types and concentrations of gases during the combustion of polyurethane and explained the fire mechanism and fire hazard of polyurethane^30^.

Many researches have shown that there are big differences between the pyrolysis and combustion of multiple system and single system^31^. During the spontaneous combustion process of coal-polyurethane binary system, there are synergistic effects, which greatly change the mechanism and gas release laws of spontaneous combustion^32–34^. However, under the existing index gas-based assessment system, there will be some serious errors in discriminating the fire situation of polyurethane-containing goafs, which will affect normal production. In this research, the dynamic mechanism and gas product release laws of the spontaneous combustion of coal-polyurethane binary system will be investigated by a combination of TGA and spontaneous combustion simulation experiments. This study is intended to provide a theoretical basis for accurately determining the spontaneous combustion situation from fire index gases.

## Experimental section

### Materials

The experimental coal samples were collected from Gaohe Coal Mine in Shanxi Province, China. The polyurethane samples were collected from the same coal mine. The polyurethane consisted of two components: black and white materials. The main component of the black material is polyisocyanate (PAPI). The main component of the white material is polyether polyol N-4110. Small amounts of auxiliary ingredients (catalysts, chain extenders, flame retardants and foam stabilizers) were included in the white material. According to the construction requirements, the samples were rigid polyurethane foams made of a 1:1 volume ratio mixture of black and white materials^31^. The coal and polyurethane samples were pulverized before the experiments and dried at 80 °C for 24 h in a nitrogen atmosphere. The proximate analysis and elemental analysis results of the samples are shown in Table 1.

**Table 1:** Proximate analysis and elemental analysis results of coal and polyurethane.

Sample	Proximate analysis				Elemental analysis				
	Mad/%	Vad/%	Aad/%	FCad/%	N/%	C/%	H/%	S/%	O/%
Coal	0.69%	15.53%	7.93%	75.86%	1.51%	83.00%	2.75%	2.77%	9.97%
Polyurethane	3.66%	88.72%	1.33%	6.29%	6.32%	60.50%	6.03%	0.00%	27.15%

### TGA experiments

The TG experiments were carried out by a NETZSCH STA-449-F3 thermal synthesis analyser. During the process of polyurethane strengthening coal rock layer, the mixing ratio of polyurethane to coal is usually lower than 1:5. For these experiments, coal was blended with polyurethane at 7 different proportions, and the samples were less than 200 mesh. The samples weighed approximately 10 mg and were heated from 30 to 800 °C at a heating rate of 2 °C/min in a synthetic air atmosphere (80 N_2_ and 20% O_2_) to simulate the spontaneous combustion process, and three repeated experiments were carried out for each sample. The samples and experimental parameters are described in Table 2.

**Table 2:** Samples and experimental conditions.

Sample	Composition	Polyurethane to coal mass ratio	Polyurethane content (%)	Heating ratio/( °C/min)
C	coal	0:1	0.00	2
PU-C-1	Polyurethane and coal	1:25	3.85	2
PU-C-2	Polyurethane and coal	1:20	4.76	2
PU-C-3	Polyurethane and coal	1:15	6.25	2
PU-C-4	Polyurethane and coal	1:10	9.09	2
PU-C-5	Polyurethane and coal	1:5	16.67	2
PU	Polyurethane	1:0	100.00	2

### Spontaneous combustion simulation experiments

**Figure 1:**
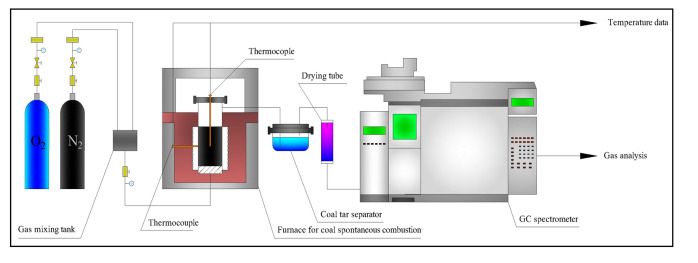
Schematic diagram of coal spontaneous combustion simulation experiment system.

In this paper, the gas release laws of coal-polyurethane binary system were researched by spontaneous combustion simulation experiments. The experimental system is shown in Fig. 1. The components and concentrations of gas produced were analysed by a XIDONG GC-4000A GC instrument (China). Seven kinds of samples of polyurethane blended with coal, with mass fraction of polyurethane of 0.00%, 3.85%, 4.76%, 6.25%, 9.09%, 16.67% and 100.00%, were selected for these experiments. Every sample was composed of 4 kinds of particles with diameters of 1 mm, 1–3 mm, 3–5 mm and 5–7 mm according to the ratio 1:1:1:1. During the experiments, the samples were heated from 20 to 500 °C with a heating rate of 2 °C/min. Each time, a 200 g sample was used, and each group of samples was analysed three times.

## Results and discussion

### TGA

**Figure 2:**
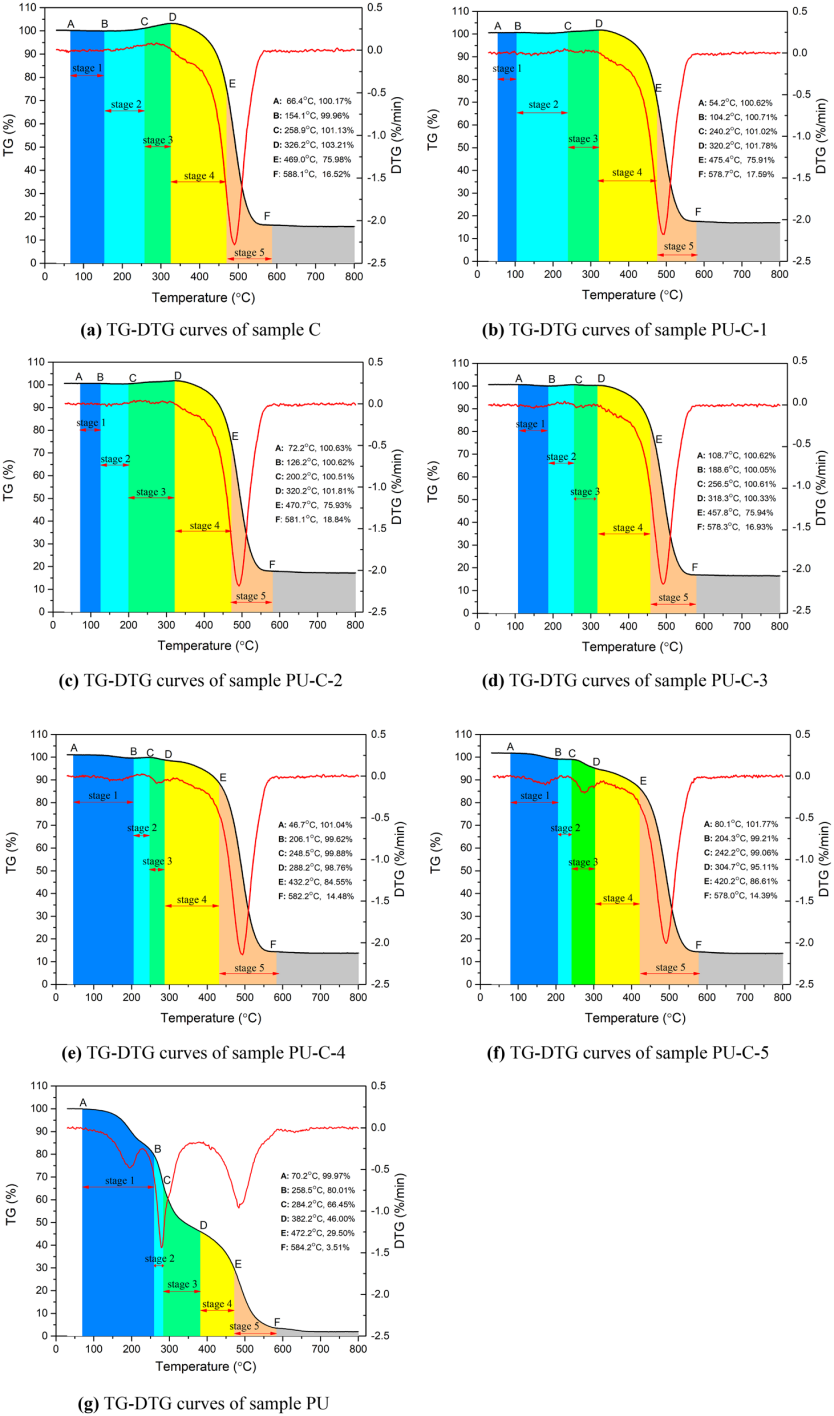
TG-DTG curves of samples.

In this paper, the double extrapolation method was used to divide the different stages of the spontaneous combustion of blended samples^35^. The TG and derivative TG (DTG) results for each sample are shown in Fig. 2, and the characteristic parameters of the spontaneous combustion process of each sample are shown in Table 3.

**Table 3:** Characteristic parameters of samples during the spontaneous combustion process.

Sample	*T*_*i*_/°C	*T*_*b*_/°C	*T*_*max*_/°C	*DTG*_*max*_/(mg/min)	*DTG*_*mean*_/(mg/min)
C	469	588.1	490.2	− 2.28	− 0.2116
PU-C-1	471.4	578.7	494.0	− 2.17	− 0.2105
PU-C-2	470.7	581.1	490.3	− 2.18	− 0.2097
PU-C-3	457.8	578.3	492.8	− 2.15	− 0.2097
PU-C-4	432.2	582.2	494.3	− 2.14	− 0.2083
PU-C-5	420.2	578.0	492.1	− 2.00	− 0.2234
PU	284.2	585.6	279.6	− 1.44	− 0.2542

*T*_*i*_ is the ignition temperature, *T*_*b*_ is the end temperature of spontaneous combustion and *T*_*max*_ is the temperature corresponding to the maximum value of DTG.

Coal is a mixture with complex physical structures and chemical compositions, and it contains a variety of organic and inorganic substances. The spontaneous combustion processes of coal are very complex. Generally, the spontaneous combustion of coal can be divided into five stages: (1) evaporation stage, (2) physical oxygen absorption stage, (3)chemical oxygen absorption stage, (4) pyrolysis stage and (5) burning stage^36^. In the evaporation stage, the moisture in the coal is evaporated by heating. During the physical oxygen absorption stage, the coal contacts air and absorbs oxygen at low temperature. During the chemical oxygen absorption stage, the active groups (–CH) in coal lose hydrogen and form carbon radicals (C^·^), and macromolecular carbon radicals (R^·^) and carbon radicals (C^·^) absorb oxygen to form peroxides (–O–O–). Then, the peroxides (–O–O–) decompose to form hydroxyl radicals (^·^OH) and ether oxygen radicals (–C–H^·^). Hydroxyl radicals (^·^OH) react with aliphatic hydrocarbons and oxygen-containing functional groups in the coal, generating free radicals and oxygen-containing free radicals. Ether oxygen radicals (–C–H^·^) react to form carbonyl (–C=O) and hydrocarbon-like gases and carbon radicals (C^·^). These new carbon radicals (C^·^) continue to undergo the abovementioned reactions and form a chain reaction cycle. During these processes, the reactions release heat continuously, and heat accumulation leads to a temperature increase; then, the reaction rate gradually accelerates^37^. In the chemical oxygen absorption stage of coal, coal pyrolysis and oxygen absorption exist together at the same time. The alkyl side chains, oxygen-containing functional groups and small-molecule groups in the coal break from the main structure of the coal, and the products escape as gaseous volatiles. At the same time, some cyclic macromolecular structures in the coal begin to break. The growth rate of oxygen-absorption groups accelerates, and the amount of oxygen absorbed is enhanced. In this stage, the rate of weight gain caused by oxygen absorption of coal is faster than the rate of pyrolysis of coal, and the overall weight of the coal increases. During the pyrolysis stage, the decomposition rate of ring-structure macromolecules in coal accelerates. The number of active structures on the surface of the coal increases rapidly, and the coal oxidation and decomposition rates accelerate. Then, a large amount of CO, CO_2_ and heat are released. During the combustion stage, the temperature reaches the ignition temperature of coal. The aromatic ring structures in the coal begin to pyrolyse, and the fixed carbon in the coal starts to burn. The pyrolysis of coal produces tars, volatiles burn violently, and large amounts of CO and CO_2_ are generated. At this time, the weight loss rate and heat release rate reach their peak values^35,36,38^.

The combustion of polyurethane can be divided into five stages, namely, the evaporation stage, pyrolysis stage, pre-charring stage, charring stage and combustion stage. During the evaporation stage, small molecules such as water and organic solvents absorbed by the polyurethane escape. During the pyrolysis stage, the C–O bonds of the amino acid methyl ester groups in the polyurethane main chains break and decompose into dicyanate and polyhydric alcohol. With increasing temperature, these compounds decompose to form amines, olefins and CO_2_. During the pre-charring stage, the main reactions are the pyrolysis of isocyanate, polyol and diinide, which release ammonia, CO_2_ and CO and produce aromatic esters that are difficult to decompose^27,39–41^. During the charring stage, the residual polyhydric alcohols, aromatic esters, cyanates and macromolecular organics are completely pyrolysed and form HCN, CO, CO_2_ and carbides. During the burning stage, the fixed carbon begins to burn and release large amounts of CO and CO_2_^28,42,43^.

The spontaneous combustion of coal-polyurethane binary system can be divided into five stages. The mass fraction of polyurethane has a great influence on the early stage of spontaneous combustion. The spontaneous combustion processes of blended samples with a mass fraction of polyurethane than 6.25% can be divided into the same stages as those of coal. When the mass fraction of polyurethane is higher than 6.25%, the spontaneous combustion process can be divided into five stages: the evaporation stage, the oxygen absorption–evaporation stage, the oxygen absorption-pyrolysis stage, the pyrolysis stage and the combustion stage. The evaporation stage of polyurethane is from 70.18 to 258.50 °C, and the physical and chemical oxygen absorption stages of coal are from 154.20 to 326.20 °C. The temperature ranges for the oxygen absorption of coal and the evaporation of polyurethane overlap. When the mass fraction of polyurethane is high enough, the combustion process of blended samples manifests as an oxygen absorption–evaporation stage. When the mass fraction of polyurethane is 3.85%3.85%, the temperature range of the physical oxygen absorption stage is the longest, 104.2–240.2 °C. This is because when the mass fraction of polyurethane is low, the evaporation of polyurethane and the physical oxygen absorption of coal inhibit each other. As a result, this stage occurs over a wider range of temperatures. As the mass fraction of polyurethane increases, the physical oxygen absorption stage becomes shorter and is replaced by an evaporation–oxygen absorption stage. This is because as the mass fraction of polyurethane increases, the evaporation of polyurethane is enhanced, corresponding to an evaporation–oxygen absorption stage. The temperature range of the pyrolysis of polyurethane is from 258.5 to 284.18 °C, and the temperature ranges of the oxygen absorption of coal and the pyrolysis of polyurethane also overlap. When the mass fraction of polyurethane increases, an oxygen absorption–pyrolysis stage appears. During this stage, the coal undergoes chemical oxygen absorption, and the polyurethane undergoes pyrolysis. The initial pyrolysis and ignition temperatures of blended samples decrease with increasing mass fraction of polyurethane because a large amount of heat and free radicals are quickly produced in polyurethane pyrolysis, which accelerates coal pyrolysis and combustion.

### Kinetic analysis of spontaneous combustion of blended samples

The reaction rate during the TG process can be expressed as follows:

1 $$ g\left( \alpha \right) = k \cdot t = \int_{0}^{\alpha } {k \cdot f\left( \alpha \right)} \cdot dt $$

2 $$ \alpha = \frac{{W_{0} - W_{t} }}{{W_{0} - W_{\infty } }} $$

where $g\left( \alpha \right)$ is the reaction mechanism integral function, $\alpha$ is the loss fraction, %, $t$ is the time, s, $k$ is the temperature-dependent constant, $f(\alpha )$ is the reaction mechanism differential function, and $W_{0}$, $W_{t}$ and $W_{\infty }$ are the initial weight, the weight at time $t$ and the weight at the end of combustion, respectively.

According to the Arrhenius equation:

3 $$ k = A \times \exp \left( {{ - }\frac{E}{RT}} \right) $$

where $A$, $E$, $T$ and $R$ are the pre-exponential factor, the apparent active energy, kJ/mol, temperature, K and the universal gas constant, respectively. By using the Coats–Redfern method to approximate the integration results, the following transformation can be obtained:

4 $$ \ln \left( {\frac{g\left( \alpha \right)}{{T^{2} }}} \right) = \ln \left( {\frac{AR}{{\beta E}}} \right) - \frac{E}{RT} $$

In the above formula, $\beta$ is the heating rate, K/min. Let $y = \ln \left[ {\frac{g\left( \alpha \right)}{{T^{2} }}} \right]$; its value can be obtained from the experimental TGA data. Let $x = \frac{1}{T}$; $y$ is linear with $x$. The slope of the equation is $k = - \frac{E}{R}$, and the intercept is $b = \ln \frac{AR}{{\beta E}}$. It can be calculated that $E = - k \cdot R$ and $A = \frac{{e^{b} \times U \times E}}{R}$. Table 4 shows the calculation formulas of common spontaneous combustion dynamics models^44–46^.

**Table 4:** General gas–solid reaction equations.

No	Reaction model	$g\left( \alpha \right)$	$f\left( \alpha \right)$
1	Zero-order	$g(\alpha ) = \alpha$	$f\left( \alpha \right) = 1$
2	First-order	$g\left( \alpha \right) = - \ln \left( {1 - \alpha } \right)$	$f(\alpha ) = 1 - \alpha$
3	Nth-order	$g(\alpha ) = \frac{1}{n - 1} \times \left[ {\left( {1 - \alpha } \right)^{ - (n - 1)} - 1} \right]$	$f\left( \alpha \right) = \left( {1 - \alpha } \right)^{n}$
4	Power law (n = 1/2)	$g(\alpha ) = \alpha^{1/2}$	$f\left( \alpha \right) = 2 \times \alpha^{1/2}$
5	Power law (n = 1/3)	$g(\alpha ) = \alpha^{1/3}$	$f\left( \alpha \right) = 3 \times \alpha^{2/3}$
6	Power law (n)	$g(\alpha ) = \alpha^{{1/{\text{n}}}}$	$f\left( \alpha \right) = {\text{n}} \times \alpha^{(n - 1)/n}$
7	Two-dimensional	$g(\alpha ) = 1 - \left( {1 - \alpha } \right)^{1/2}$	$f\left( \alpha \right) = 2 \times \left( {{1 - }\alpha } \right)^{{1/2}}$

In this paper, the Malek method is used to infer the kinetic mechanism function for the spontaneous combustion of different samples^46,47^. This method defines the function as follows:

5 $$ y\left( \alpha \right) = \left( {\frac{T}{{T_{0.5} }}} \right)^{2} \cdot \frac{{\left( {\frac{d\alpha }{{dt}}} \right)}}{{\left( {\frac{d\alpha }{{dt}}} \right)_{0.5} }} = \frac{f\left( \alpha \right) \cdot g\left( \alpha \right)}{{f\left( {0.5} \right) \cdot g\left( {0.5} \right)}} $$

The experimental data $$\alpha_{i}$$, $$T_{i}$$, $$\left( {\frac{d\alpha }{{dt}}} \right)_{i}$$$$\left( {i = 1,2, \cdots ,j} \right)$$, $$\alpha_{0.5}$$, $$T_{0.5}$$ and $$\left( {\frac{d\alpha }{{dt}}} \right)_{0.5}$$ can be inserted into function (5). The curve of $$y\left( \alpha \right) - \alpha$$ can be regarded as the experimental curve. If the experimental curve overlaps with the standard curve, the function corresponding to the standard curve is determined to be the most probable dynamic mechanism function.

In this paper, the most probable mechanism functions in different stages of the spontaneous combustion process of blended samples are judged by the Malek method. The stages of spontaneous combustion for the samples and their corresponding reaction model number, curve thermodynamic equation, activation energy and pre-exponential factor are shown in Table 5.

**Table 5:** Activation energy, pre-exponential factor and fitting kinetic equation for each stage of the blended samples.

Sample	Stage	Kinetic function model	Fitting equation	R^2^	E/(kJ/mol)	A/min^−1^
C	Evaporation stage	No. 3	y = − 3326.92x − 17.865	0.9374	27.66	1.16E−04
	Physical oxygen absorption stage	–	–	–	–	–
	Chemical oxygen absorption stage	No. 3	y = − 7646.14x − 4.6634	0.9571	63.57	1.90E+02
	Pyrolysis stage	No. 6, n = 1.2	y = − 17,305.75x + 15.525	0.9748	143.88	2.43E+11
	Burning stage	No. 6, n = 1.2	y = − 13,917.49x + 11.139	0.9839	115.71	2.58E+09
PU-C-1	Evaporation stage	No. 3	y = − 3917.49x − 9.6642	0.9043	32.57	8.19E−01
	Physical oxygen absorption stage	–	–	–	–	–
	Chemical oxygen absorption stage	No. 3	y = − 9413.04x − 13.033	0.9777	78.26	2.68E-02
	Pyrolysis stage	No. 6, n = 1.5	y = − 18,331.73x + 7.4284	0.9899	152.41	1.50E+10
	Burning stage	No. 6, n = 1.5	y = 13,791.20x + 18.29	0.9945	114.66	4.23E+12
PU-C-2	Evaporation stage	No. 3	y = − 4337.26x − 9.6115	0.9308	36.06	2.30E+08
	Physical oxygen absorption stage	–	–	–	–	–
	Chemical oxygen absorption stage	No. 3	y = − 11,092.13x − 14.108	0.9725	92.22	8.33E-03
	Pyrolysis stage	No. 6, n = 1.5	y = − 19,836.42x + 7.7157	0.9914	164.92	7.32E+07
	Burning stage	No. 6, n = 1.5	y = − 13,904.26x + 18.101	0.9945	115.6	3.48E+12
PU-C-3	Evaporation stage	No. 6, n = 1.2	y = − 4663.22x − 0.8552	0.9505	38.77	6.11E+03
	Evaporation–oxygen absorption stage	–	–	–	–	–
	Oxygen absorption-pyrolysis stage	No. 6, n = 1.2	y = − 7490.98x + 4.8143	0.9813	62.28	1.27E+06
	Pyrolysis stage	No. 6, n = 1.2	y = − 15,769.79x + 1.2496	0.9952	131.11	8.07E+04
	Burning stage	No. 6, n = 1.2	y = − 14,087.08x + 14.463	0.9989	117.12	8.12E+10
PU-C-4	Evaporation stage	No. 6, n = 1.3	y = − 4797.93x − 8.4171	0.9459	39.89	3.36E+07
	Evaporation-oxygen absorption stage	–	–	–	–	–
	Oxygen absorption-pyrolysis stage	No. 6, n = 1.3	y = − 7083.23x − 7.9301	0.9846	58.89	3.37E+00
	Pyrolysis stage	No. 6, n = 1.3	y = − 14,218.19x − 8.0256	0.9885	118.21	3.24E+00
	Burning stage	No. 6, n = 1.3	y = − 14,049.80x + 11.269	0.9937	116.81	2.93E+09
PU-C-5	Evaporation stage	No. 3	y = − 5158.77x − 5.6533	0.9900	42.89	3.28E+01
	Evaporation-oxygen absorption stage	–	–	–	–	–
	Oxygen absorption-pyrolysis stage	No. 3	y = − 6609.33x − 8.3237	0.9822	54.95	1.92E+00
	Pyrolysis stage	No. 6, n = 1.2	y = − 13,491.70x − 11.517	0.9786	112.17	4.54E+08
	Burning stage	No. 6, n = 1.2	y = − 14,219.39x + 5.5117	0.9910	118.22	7.10E+06
PU	Evaporation stage	No. 6 n = 1.5	y = − 6695.6x + 24.102	0.9930	55.67	3.93E+14
	Pyrolysis stage	No. 6, n = 1.5	y = − 8703.7x + 27.468	0.9905	72.36	1.48E+16
	Pre-charring stage	No. 6, n = 1.5	y = − 3543.5x + 18.426	0.9650	29.46	7.12E+11
	Charring stage	No. 6, n = 1.5	y = − 4239.9x + 19.361	0.9705	35.25	2.17E+12
	Burning stage	No. 6, n = 1.5	y = − 14005x + 32.581	0.9976	116.44	3.95E+18

The number of kinetic function of each stage shows in Table 4.

Coal, polyurethane and blended samples have different kinetic functions at different stages of spontaneous combustion. The dominating chemical reactions during each stage of the spontaneous combustion process of different samples are different. During the evaporation stage, the moisture and organic solvents in the samples are heated and volatilized. There are almost no chemical reactions. Only moisture is present in coal, and the activation energy of the coal evaporation stage is the lowest, 27.66 kJ/mol. Polyurethane contains moisture and organic solvents, and the activation energy of the polyurethane evaporation stage is the greatest, 55.67 kJ/mol. The activation energy of the evaporation stage of blended samples increases as the mass fraction of polyurethane rises. During the physical oxygen absorption and evaporation–oxygen absorption stages, the TG curves change little and fluctuate greatly. Therefore, the activation energy cannot be calculated accurately. The activation energies of the chemical oxygen absorption stage and oxygen absorption-pyrolysis stage are significantly influenced by the mass fraction of polyurethane. When the mass fraction of polyurethane is less than 6.25%, the chemical oxygen-absorption stage corresponds to weight gain, and the activation energy of these blended samples is higher than that of coal. When the mass fraction of polyurethane is low, the oxygen absorption of coal dominates and competes with the pyrolysis of polyurethane. These two reactions inhibit each other, which leads to an increase in activation energy. When the mass fraction of polyurethane is higher than 6.25%, the processes in blended samples manifest as weight loss, and the activation energy of these processes is lower than that of coal. When the mass fraction of polyurethane is high, the pyrolysis of polyurethane dominates, and heat and free radicals are released. This reaction replaces chemical oxygen absorption to produce heat and free radicals. As a result, the activation energy of these processes decreases. The activation energy change law of the pyrolysis stage is the same as that of the oxygen absorption-pyrolysis stage. When the mass fraction of polyurethane is lower than 6.25%, the activation energy is higher than that of coal. When the mass fraction of polyurethane is higher than 6.25%, the activation energy is lower than that of coal. Combustion is a chemical reaction process controlled jointly by energy and free radicals. When the mass fraction of polyurethane is low, a small amount of polyurethane pyrolyses and oxidizes to produce a small amount of heat and free radicals, which inhibits the pyrolysis of coal, and the activation energy of these processes increases. When the mass fraction of polyurethane is higher than 6.25%, the pyrolysis and oxidation of polyurethane release large amounts of heat and radicals, which promote the pyrolysis of coal, and the activation energy of these processes decreases. The activation energies of the burning stages of blended samples and coal are nearly the same. Because the pyrolysis products of polyurethane and coal are both fixed carbon, during these stages, there are few differences among the reactions of coal, polyurethane and blended samples, and the activation energies of different samples are nearly equal.

### Morphology changes in samples after spontaneous combustion

**Figure 3:**
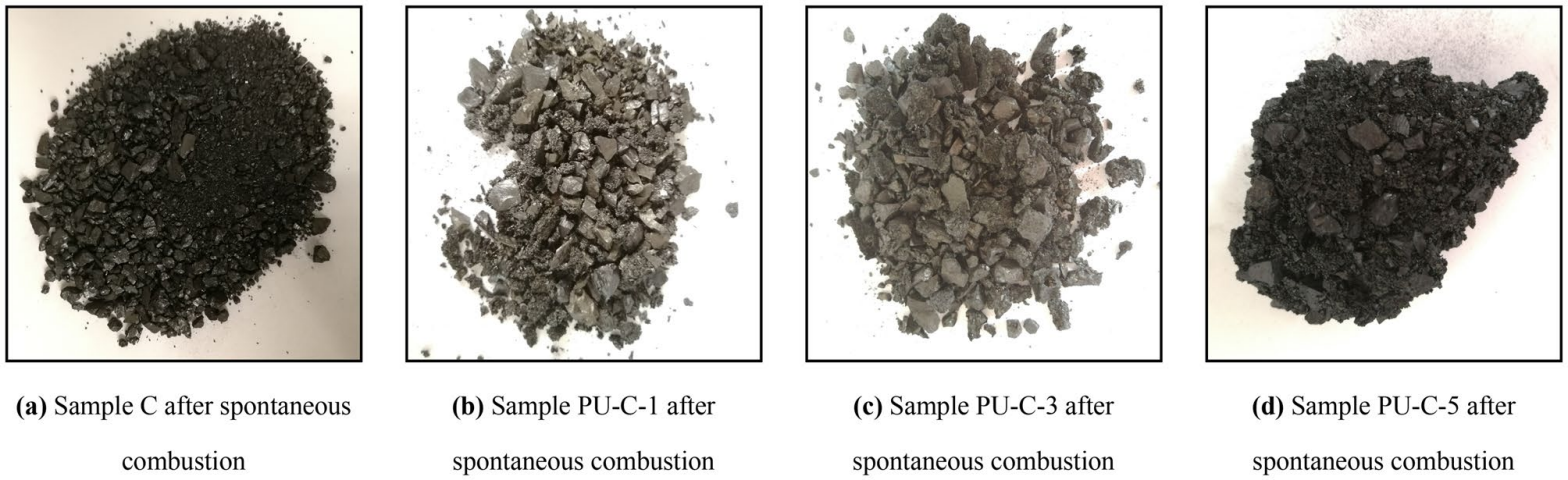
Different samples after spontaneous combustion.

**Figure 4:**
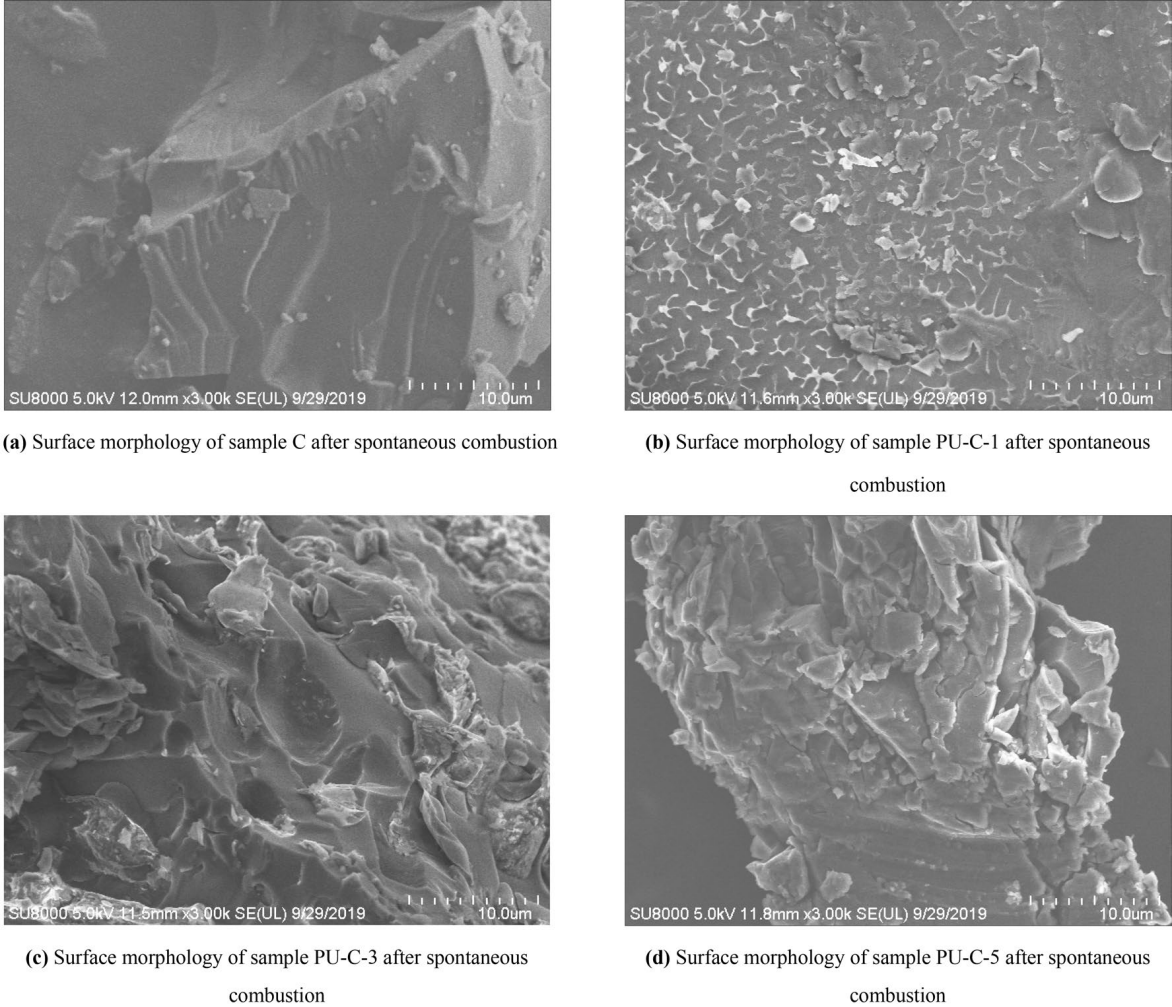
Surface morphology of different samples after spontaneous combustion.

The morphological structure and coking state of blended samples before and after the spontaneous combustion experiments changed significantly, as shown in Fig. 3. Scanning electron microscopy (SEM) was used to observe the surface structures of the mixed samples. The surface structures of the blended samples are shown in Fig. 4.

After spontaneous combustion, the surface gloss of coal disappears, and the sample particles are loose and do not undergo coking. After spontaneous combustion, the morphologies of blended samples change with mass fraction of polyurethane. Some loose porous particles, which are the residues of polyurethane melting and burning, appear in the blended samples. As the mass fraction of polyurethane increases, the number of porous particles increases, and the residues become coked. The loose porous particles in the residues after spontaneous combustion are the carbon skeletons of polyurethane after the charring process. Polyurethane is a noncrystalline polymer with a complex composition and no fixed melting temperature. During combustion, its pyrolysis, melting, charring and burning processes overlap with each other.

The surface microscopic morphologies of coal particles after spontaneous combustion are shown in Fig. 4a. The surfaces of the coal particles are flat and smooth, and there are a few cracks and almost no small particles attached to the surface. The surface microscopic morphology of the sample with a mass fraction of polyurethane of 3.85% (sample PU-C-1) after spontaneous combustion is shown in Fig. 4b. The surface is rough, with many regular extended textured features that are the traces left by the melting and burning of polyurethane. The surface microscopic morphology of the sample with a mass fraction of polyurethane of 6.25% (sample PU-C-3) is shown in Fig. 4c. The surface structure is much rougher than that of coal, and there are many irregular depressions and protrusions. The pyrolysis and combustion of polyurethane particles release a large amount of heat, which accelerates the pyrolysis and combustion of the surface of the coal particles in contact with them. Then, some depressions form on the surface of the particles. The protrusions on the surface of the particles are caused by the combustion residues of polyurethane left on them after cooling. The surface microscopic morphology of the sample with a mass fraction of polyurethane of 16.67% (sample PU-C-5) is shown in Fig. 4d. Many combustion residues are stuck to the coal surface, making the surface of the coal much rougher, and there are more cracks and small particles on the surface. Therefore, blending with polyurethane changes the surface morphology of coal after spontaneous combustion. It makes the surface of coal particles rougher, increases their specific surface area and promotes their combustion.

### Gas release laws during spontaneous combustion

**Figure 5:**
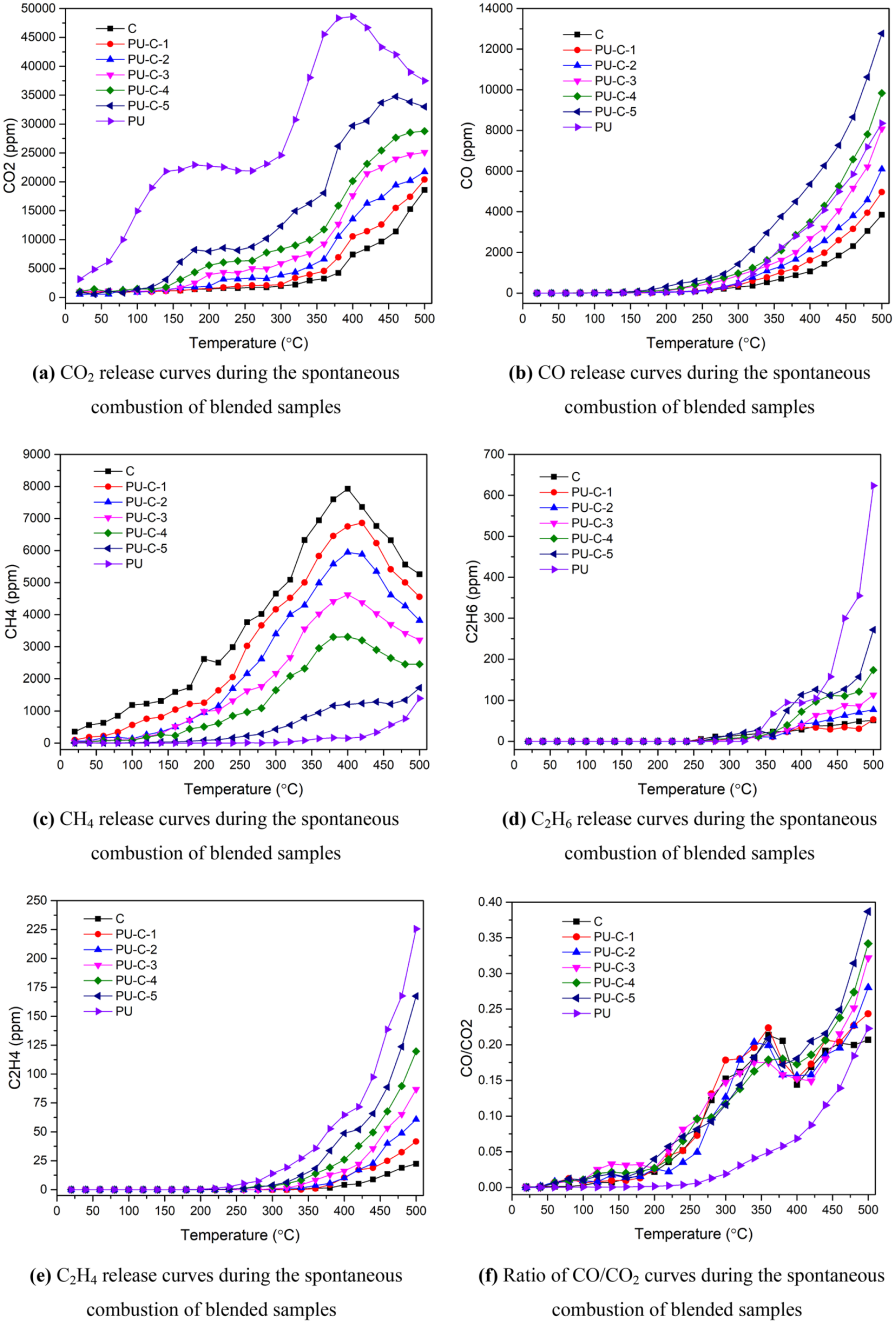
Curves of fire gas release during the spontaneous combustion of blended samples.

The fire gas release curves of coal, polyurethane and blended samples determined during spontaneous combustion simulation experiments are shown in Fig. 5.

### Release laws of carbon oxide gases

1. Release laws of CO_2_. During the spontaneous combustion of coal, the initial concentration of CO_2_ is 865.47 ppm. From 20 to 320 °C, the CO_2_ concentration rises slowly. At 360 °C, the CO_2_ concentration begins to increase rapidly and reaches a maximum of 18,594.00 ppm at 500 °C. Before 360 °C, the main reaction of coal is oxygen absorption, and its pyrolysis is relatively weak, which leads to a lower yield of CO_2_. When the temperature reaches 360 °C, the pyrolysis of coal accelerates, and more carbonyl and hydroxyl groups in the coal break to produce CO_2_.
  During the combustion of polyurethane, the initial concentration of CO_2_ is 3167.29 ppm, which is higher than that of coal. From 20 °C, the CO_2_ concentration increases rapidly and reaches 21,830 ppm at 140℃. From 140 to 280 °C, the CO_2_ concentration remains stable. From 280 to 400 °C, the CO_2_ concentration rises rapidly again and reaches a maximum at 400 °C of 48,611.64 ppm. From 400 to 500 °C, the CO_2_ concentration drops to 37,491.30 ppm. In the polyurethane synthesis process, isocyanate and polyhydric alcohol react to form CO_2,_ which is stored in the pores of the polyurethane. After spontaneous combustion starts, the moisture, organic solvent and residual CO_2_ in the polyurethane desorb, and the CO_2_ concentration increases. From 140 to 280 °C, the main reaction is the depolymerization of polyurethane, and almost no CO_2_ is generated. At 280 °C, the pre-charring stage of polyurethane begins, and the concentration of CO_2_ rises rapidly. When the temperature reaches 400 °C, the main reaction is the combustion of the residual fixed carbon from polyurethane pyrolysis. Due to the insufficient oxygen supply, the combustion is incomplete, and the CO_2_ concentration drops.
  During the spontaneous combustion of blended samples, the CO_2_ concentration is significantly affected by the mass fraction of polyurethane. When the temperature is higher than 120 °C, the CO_2_ concentration at a given temperature of the blended samples increases with the mass fraction of polyurethane. The CO_2_ concentration change laws of blended samples with a mass fraction of polyurethane higher than 6.25% are similar to that of polyurethane; the CO_2_ concentration increases significantly after 200 °C and remains stable from 200 to 280 °C. From 280 to 460 °C, the CO_2_ concentration increases rapidly. After 460 °C, the CO_2_ concentration drops or remains stable. When the mass fraction of polyurethane is less than 6.25%, the CO2 concentration change law is similar to that of coal. When coal is blended with polyurethane, the CO_2_ concentration rises during spontaneous combustion compared to that of coal. This is because the pyrolysis of polyurethane produces large amounts of free radicals and CO_2_, and the activation energy of each stage of the combustion of polyurethane is lower than that of coal. The pyrolysis of polyurethane in the blended samples provides free radicals for the pyrolysis and combustion of coal. With increasing mass fraction of polyurethane, the combustion of blended samples accelerates, and the CO_2_ concentration rises.
2. Release laws of CO. During the spontaneous combustion of coal, the CO concentration increases with increasing temperature. At 200 °C, the CO concentration exceeds 24 ppm. From 320 to 500 °C, the CO concentration rises rapidly and reaches a maximum of 3848.60 ppm. When the temperature is lower than 320 °C, the main reaction is the oxygen absorption of coal, and the pyrolysis of coal is weak. After 320 °C, the pyrolysis of coal is enhanced. During this stage, there are two main reactions. One reaction is the cleavage of carboxyl groups and carbonyl groups, and the other is CO_2_ reacting with coal to produce CO. The CO concentration increases rapidly.
  During the combustion of polyurethane, the CO concentration increases with increasing temperature. At 180 °C, the CO concentration exceeds 24 ppm. From 280 to 500 °C, the CO concentration increases rapidly and reaches a maximum of 8356.60 ppm. The pre-charring stage of polyurethane starts at 320 °C. During this stage, isocyanate, amides and oxygen-containing functional groups break to produce CO. At the same time, the volatiles generated by the pyrolysis of polyurethane burn and release CO. As the temperature increases, the production rate of CO accelerates.
  During the spontaneous combustion of blended samples, the mass fraction of polyurethane has a significant influence on the CO concentration. As the mass fraction of polyurethane increases, the temperature at which the CO concentration abruptly starts to rapidly increase becomes lower, and the CO concentration at a given temperature is higher. The temperature at which the CO concentration abruptly changes in sample PU-C-5 is 160 °C. The CO concentration in the spontaneous combustion of blended samples with a mass fraction of polyurethane less than 6.25% is higher than that of coal and lower than that of polyurethane. When the mass fraction of polyurethane is higher than 6.25%, the CO concentration of blended samples is higher than those of coal and polyurethane, and the maximal CO concentration of sample PU-C-5 is 12,767.66 ppm. There are synergistic effects during the spontaneous combustion of blended samples. The pyrolysis and combustion processes of polyurethane provide heat and free radicals to coal, which accelerates the pyrolysis and combustion of coal. The pyrolysis and oxidation of polyurethane consume more oxygen than the corresponding reactions of coal. The incomplete combustion of coal-polyurethane binary system accelerates the generation of CO. Moreover, the polyurethane in blended samples melts as the temperature rises, and the combustion of blended samples scorches the particles of coal and polyurethane into lumps. The cracks in blended samples enable air flow, which promotes the burning of the surface of sample blocks. The combustion inside the sample blocks is incomplete, and the CO concentration increases. Therefore, the spontaneous combustion of coal-polyurethane binary system promotes the formation of CO.
  The curves of the CO/CO_2_ ratio during the spontaneous combustion of blended samples are shown in Fig. 5f. The CO/CO_2_ ratio curves of coal and blended samples are almost the same, and the CO/CO_2_ ratio curve of polyurethane is lower than those of other samples. CO_2_ production during the spontaneous combustion of polyurethane is much higher than that during the spontaneous combustion of other samples. The main reactant in coal samples and blended samples is coal. Therefore, the CO/CO_2_ ratios of coal and blended samples are similar at the same temperature. From 20 to 350 °C, the CO/CO_2_ ratio rises with increasing temperature. From 350 to 420 °C, the CO/CO_2_ ratio decreases slightly. From 420 to 500 °C, the ratio increases again. This trend occurs because from 350 to 420 °C, the pyrolysis and violent oxidation of coal release a large amount of CO_2,_ and the CO/CO_2_ ratio decreases. As pyrolysis and oxidation proceed, the samples begin to burn, and the oxygen supply is insufficient. Then, the generation of CO increases, the generation of CO_2_ decreases, and the CO/CO_2_ ratio drops. The CO/CO_2_ ratios of coal and blended samples are independent of the mass fraction of polyurethane. The CO/CO_2_ ratio has a good correlation with spontaneous combustion temperature, meaning that this parameter can describe the situation of spontaneous combustion more accurately than the existing index gas method.

### Release laws of hydrocarbon gases

1. Release laws of CH_4_. During the spontaneous combustion of coal, the CH_4_ concentration first increases and then decreases. From 20 to 400 °C, the CH_4_ concentration rises with increasing temperature and reaches a maximum of 7928.51 ppm at 400 °C. Then, from 400 to 500 °C, the CH_4_ concentration drops rapidly. The coal used in the experiments is bituminous coal, which has a high degree of coalification, and a certain amount of CH_4_ exists in the pores of coal. During the spontaneous combustion process, as the temperature rises, CH_4_ desorbs from the coal. At 400 °C, the desorption of CH_4_ ends, CH_4_ begins to oxidize, and the CH_4_ concentration decreases.
  During the combustion of polyurethane, the CH_4_ concentration increases with increasing temperature. At 500 °C, the CH_4_ concentration reaches 1395.18 ppm. There is no CH_4_ in the pores of polyurethane. As the temperature rises, the polyurethane begins to pyrolyse, and the methyl groups on the macromolecular branches in polyurethane break to form CH_4_. Then, the CH_4_ concentration increases.
  During the spontaneous combustion of blended samples, the CH_4_ concentration at a given temperature decreases with increasing mass fraction of polyurethane. The CH_4_ concentration change laws of blended samples with a mass fraction of polyurethane is less than 9.09% are similar to that of coal. At 400 °C, the CH_4_ concentration reaches a maximum and then begins to decrease. The higher the mass fraction of polyurethane is, the lower the maximum CH_4_ concentration is. The change trend of the CH_4_ concentration of blended samples with a mass fraction of polyurethane of 16.67% is close to that of polyurethane. During the spontaneous combustion process, the coal samples remain loose. As the temperature rises, the polyurethane in the blended samples melts and scorches with coal, and the particles of coal are coked into lumps. The cracks between particles are blocked, which inhibits the desorption of CH_4_. Therefore, coal-polyurethane binary system inhibits the generation and desorption of CH_4_.
2. Release laws of C_2_H_6_. During the spontaneous combustion of coal, C_2_H_6_ begins to release at 260 °C, and the concentration of C_2_H_6_ rises with increasing temperature. At 500 °C, the C_2_H_6_ concentration reaches a maximum of 51.06 ppm.
  During the combustion of polyurethane, C_2_H_6_ begins to release at 340 °C. As the temperature increases, the C_2_H_6_ concentration rises rapidly. At 500 °C, it reaches a maximum of 623.65 ppm.
  During the spontaneous combustion of blended samples, C_2_H_6_ begins to release at 260 °C. The C_2_H_6_ concentration of different blended samples at a given temperature rises with increasing mass fraction of polyurethane. The ethyl groups in the macromolecular structure of coal and polyurethane break to produce ethane. Some small molecular structures in coal gradually decompose to ethane after 200 °C. Polyurethane is pyrolysed into isocyanate and polyhydric alcohol first and then further cracked into small molecular substances after 300 °C. These small molecules pyrolyse produce C_2_H_6_. During the combustion of polyurethane, the temperature at which C_2_H_6_ begins to release is higher, and more C_2_H_6_ is produced. From 300 to 500 °C, the activation energy of the spontaneous combustion of blended samples decreases as the mass fraction of polyurethane increases, and the pyrolysis of samples is easier. Therefore, v promotes the formation of C_2_H_6_.
3. Release laws of C_2_H_4_. During the spontaneous combustion of coal, the concentration of C_2_H_4_ rises with increasing temperature. At 500 °C, the concentration of C_2_H_4_ reaches a maximum of 22.36 ppm.
  During the combustion of polyurethane, C_2_H_4_ begins to release at 200 °C. The C_2_H_4_ concentration rises with increasing temperature and reaches a maximum of 255.54 ppm at 500 °C.
  During the spontaneous combustion of blended samples, the temperature of at which C_2_H_4_ is first released drops as the mass fraction of polyurethane increases. The C_2_H_4_ concentration at a given temperature rises with increasing mass fraction of polyurethane. At 300 °C, the unsaturated macromolecular bonds in the coal decompose to produce ethylene. Polyurethane contains more unsaturated bonds, and the pyrolysis temperature of polyurethane is lower than that of coal. From 300 to 500 °C, the activation energy of the spontaneous combustion of blended samples drops as the mass fraction of polyurethane increases, and the samples are easier to pyrolyse and burn. Therefore, coal-polyurethane binary system promotes the formation of C_2_H_4_.
  In summary, during the spontaneous combustion of coal-polyurethane binary system, the initial release temperatures of CO_2_, CO, C_2_H_6_ and C_2_H_4_ decrease as the mass fraction of polyurethane increases. The concentration of these gases at a given temperature rises with increasing mass fraction of polyurethane, and the CH_4_ concentration drops as the mass fraction of polyurethane increases. Polyurethane promotes the generation of fire gases during the spontaneous combustion of coal, and it makes the release temperature of fire gases drop and their concentration increase. Therefore, when spontaneous combustion occurs in polyurethane-containing coal seams or goafs, there will be some errors in assessments of the spontaneous combustion temperature and situation based on the index gases of CO_2_, CO, C_2_H_6_ and C_2_H_4_. The ratios of CO/CO_2_ during the spontaneous combustion of coal samples and blended samples have the same change trends with respect to temperature. Using the CO/CO_2_ ratio as the judgement index can effectively reduce the error.

## Conclusions

1. The mass fraction of polyurethane changes the oxygen absorption stage and pyrolysis stage of spontaneous combustion processes of coal-polyurethane binary system. When the mass fraction of polyurethane is lower than 6.25%, polyurethane inhibits the oxygen absorption and pyrolysis of coal, and the activation energies of the oxygen absorption stage and pyrolysis stage are higher than those of coal. When the mass fraction of polyurethane is higher than 6.25%, polyurethane promotes the spontaneous combustion of coal. The pyrolysis temperature and ignition temperature of blended samples decrease as the mass fraction of polyurethane rises. The activation energies of the oxygen absorption-pyrolysis stage and pyrolysis stage decrease as the mass fraction of polyurethane rises, which makes spontaneous combustion easier.
2. During spontaneous combustion, the sample particles of coal-polyurethane binary system scorch into lumps. The higher the mass fraction of polyurethane is, the larger the coking lumps are. SEM observation shows that the surface of blended sample particles is covered with polyurethane combustion residues. When the mass fraction of polyurethane is low, obvious textured features indicating polyurethane residues on the coal particles are present. When the mass fraction of polyurethane is high, the surface of the coal particles is rough and irregular. Polyurethane increases the specific surface area of samples and promotes spontaneous combustion.
3. During the spontaneous combustion of coal-polyurethane binary system, the mass fraction of polyurethane has a significant influence on index gas release laws. The initial release temperature of the index gases CO, CO2, C_2_H_6_ and C_2_H_4_ drops as the mass fraction of polyurethane increases. The CH_4_ release amount drops as the mass fraction of polyurethane increases. The trends of the CO/CO_2_ ratio with respect to temperature of blended samples of different mass fraction of polyurethanes are almost the same.
